# The complete mitochondrial genome of *Zaomma eriococci* (hymenoptera: encyrtidae)

**DOI:** 10.1080/23802359.2024.2351539

**Published:** 2024-06-12

**Authors:** Zi-Cong Li, Arong Luo, Qing-Song Zhou, Zhulidezi Aishan

**Affiliations:** a Xinjiang Key Laboratory of Biological Resources and Genetic Engineering, College of Life Science and Technology, Xinjiang University, Urumqi, China; b Key Laboratory of Zoological Systematics and Evolution, Institute of Zoology, Chinese Academy of Sciences, Beijing, China

**Keywords:** *Zaomma eriococci*, encyrtidae, mitogenome

## Abstract

The complete mitochondrial genome of the *Zaomma eriococci* (Ferrière, 1955) (Hymenoptera: Encyrtidae) was obtained through next-generation sequencing, making the first reported complete mitochondrial genome of the genus *Zaomma*. The mitochondrial genome is 15,648 bp in length and includes 37 classical eukaryotic mitochondrial genes along with an A + T rich region. All 13 protein-coding genes (PCGs) initiate with typical ATN codons. Of these, 10 PCG genes terminate with TAA, while three terminate with TAG. Additionally, there are 22 tRNA genes, ranging in size from 62 to 70 bp. The maximum likelihood phylogenetic tree was constructed based on 13 PCGs, indicates that *Z. eriococci* is closely related to *Tassonia gloriae*. This mitochondrial genome will serve as a valuable molecular resource for species identification, genetic analysis, and comparative genomic studies of *Z. eriococci*, contributing to the growing collection of mitochondrial genomes within the family Encyrtidae.

## Introduction

The family Encyrtidae (Hymenoptera: Chalcidoidea) is one of the most diverse groups of parasitoids, with approximately 5,000 described species (Noyes 2018). The genus *Zaomma* was initially characterized by Ashmead in 1900 due to its very large rounded eyes and short antennae (Ashmead 1900), and presently encompasses 17 described species (Noyes 2018). *Zaomma eriococci* (Ferrière, 1955) is widely distributed in Europe and Asia, where it acts as a parasitoid for 13 scale insect species, most of which belong to the family Eriococcidae (Noyes 2018). Additionally, *Z eriococci* has been reported as a hyperparasitoid of two encyrtid species (Xu et al. 1996).

Recently, *Z. eriococci* was reported as one member of the parasitoid communities associated with Crapemyrtle Bark Scale *Acanthococcus lagerstroemiae* in China and Korea (Zhang et al. 2018; Suh 2019). However, only partial COI gene data are available for the molecular information of *Z. eriococci* (Zhang et al. 2018). The absence of complete mitochondrial genome information hinders our comprehensive understanding of this species and the phylogenetic relationships within the genus *Zaomma*, which belongs to the family Encyrtidae. In this study, we present the complete mitochondrial genome of *Z. eriococci*.

## Materials and methods

### Sampling and identification

**Figure 1.**
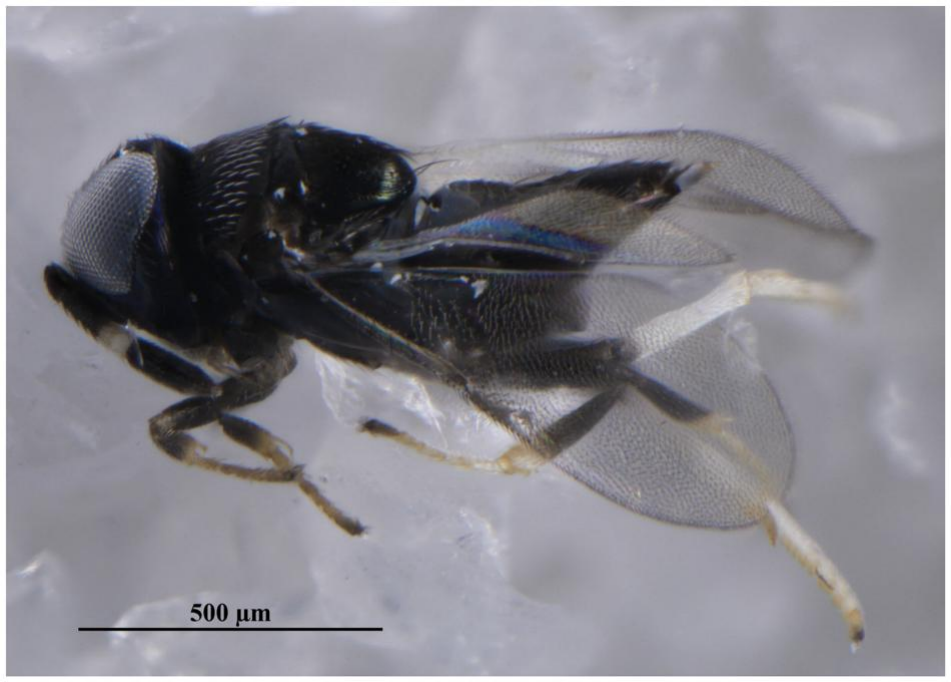
The lateral view of *Zaomma eriococci* (Ferrière, 1955). (Photo by Jin-Ling Wang). The specimen was rared from *Acanthococcus lagerstroemiae* at Jincheng City, Shanxi Province, China (112.91367E, 35.62242 N).

Specimens of *Z. eriococci* (Figure 1) were reared from *A. lagerstroemiae* (Hemiptera: Eriococcidae) collected in August 2023 in Jincheng City, Shanxi Province, China (112.91367E, 35.62242 N). The species was identified based on morphological characters as described (Suh 2019) and later confirmed by comparing the DNA barcodes with the GenBank database. Voucher specimens were preserved in 100% ethanol at −20 °C and deposited at the Institute of Zoology, Chinese Academy of Sciences (IZCAS) under voucher number J23094_TXF. For further information, please contact Qing-Song Zhou *via* email at zhouqingsong@ioz.ac.cn.

### Mitochondrial genome assembly and annotation

The complete mitochondrial genome of *Z. eriococci* was obtained through next-generation sequencing. Genomic DNA was extracted from a mixture of fresh samples from five individuals using a modified CTAB procedure in liquid nitrogen (Doyle 1986). Subsequently, DNA quality was assessed using PicoGreen and quantified by the ND-2,000 (NanoDrop Technologies). DNA integrity was confirmed by agarose gel electrophoresis. A DNA library was created using the Illumina TruSeq^®^ DNA PCR-Free HT Kit and sequenced on the Illumina HiSeq platform (150 bp paired-end). The minimum and maximum reads mapping depths for assembled mitochondrial genome were 870× and 7995×, respectively (Supplementary Figure S1). The mitochondrial genome of *Z. eriococci* were assembled from Illumina short reads using MitoFlex (Li et al. 2021) and NOVOPlasty (Dierckxsens et al. 2017). The assembled linear contigs were manually curated in BioEdit (Hall et al. 2011) and the identical sequence overlap in the control region was deleted to ensure the circularization of the mitochondrial genome.

The mitochondrial genome was annotated using the Mitos webserver (http://mitos2.bioinf.uni-leipzig.de/index.py) (Bernt et al. 2013) following the invertebrate mitochondrial code. Transfer RNA (tRNA) genes were confirmed using the online tool ARWEN (http://130.235.46.10/ARWEN/) (Laslett and Canbäck 2008). Subsequently, the position and direction of each gene were manually checked with reference mitochondrial genomes from closely related species to *Z. eriococci*. The circular map of the mitochondrial genomes was generated using OrganellarGenomeDRAW (OGDRAW, https://chlorobox.mpimp-golm.mpg.de/OGDraw.html) (Lohse et al. 2007).

### Phylogenetic analysis

A total of 16 species from the family Encyrtidae were selected as ingroups, with two species from Aphelinidae chosen as outgroups (Supplementary Table S1). The mitochondrial sequences were processed using PhyloSuite (Zhang et al. 2020). Protein coding sequences alignment were performed using MACSE v2 (Ranwez et al. 2018). Gaps and ambiguous sites were removed using Gblocks (Talavera and Castresana 2007). The phylogenetic tree was inferred using IQ-Tree v1.6.9 (Nguyen et al. 2015) under maximum-likelihood (ML) method with 1,000 ultrafast bootstraps (Hoang et al. 2018) and 1,000 SH-aLRT replicates (Guindon et al. 2010), the best-fit partitioning schemes and substitution models were determined using ModeFinder (Kalyaanamoorthy et al. 2017).

## Results

**Figure 2.**
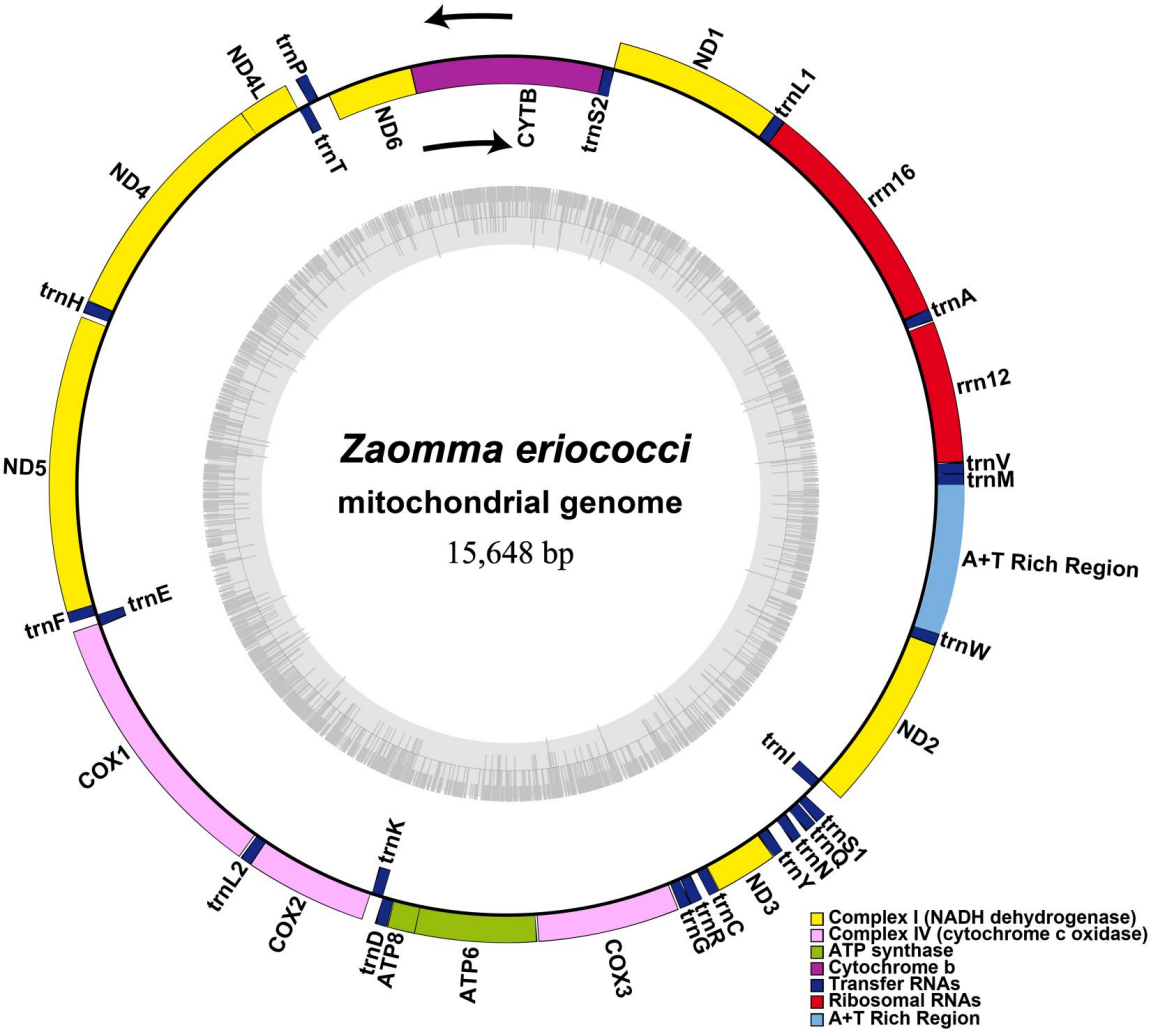
Circular map of the *Z. eriococci* mitochondrial genome. Different colors indicate different types of genes and regions.

The complete mitochondrial genome of *Z. eriococci* is a typical circular DNA molecule of 15,648 bp in length, with the nucleotide base composition as follows: A: 34.94%, T: 44.93, G: 13.43, C: 6.70%. It comprises 13 protein-coding genes (PCGs), 22 transfer RNAs (tRNAs), two ribosomal RNAs (rRNAs) and a putative control region (CR) (Figure 2). The mitochondrial genome of *Z. eriococci* exhibits a novel gene rearrangement in encyrtids species (Xing et al. 2022), a total of 29 genes, including eight PCGs, nineteen tRNAs, and two rRNAs were rearranged compared to the putative ancestral insect mitochondrial genome (Cameron 2014). The combined length of all 13 protein-coding genes accounts for 70.6% of the entire mitochondrial genome. The ND5 domain, spanning 1,662 bp, was the longest gene, while the ATP8 domain, covering 159 bp, was the shortest. All 13 PCGs initiate with typical ATN codons (six ATT, four ATG, two ATA, and one ATC). Regarding termination codons, COX2, ND1, and ND3 concluded with TAG, while the others conclude with TAA. The control region is 822 bp in length and contains seven types of repeats ranging from 10 to 144 bp. Two novel lncRNAs along with multiple copy number variations in short tandem repeats were found in other animal mitochondrial genomes (Gao et al. 2018; Chen et al. 2020), however, they were not taken into consideration in this study due to the limitations of NGS sequencing (Tørresen et al. 2019).

**Figure 3.**
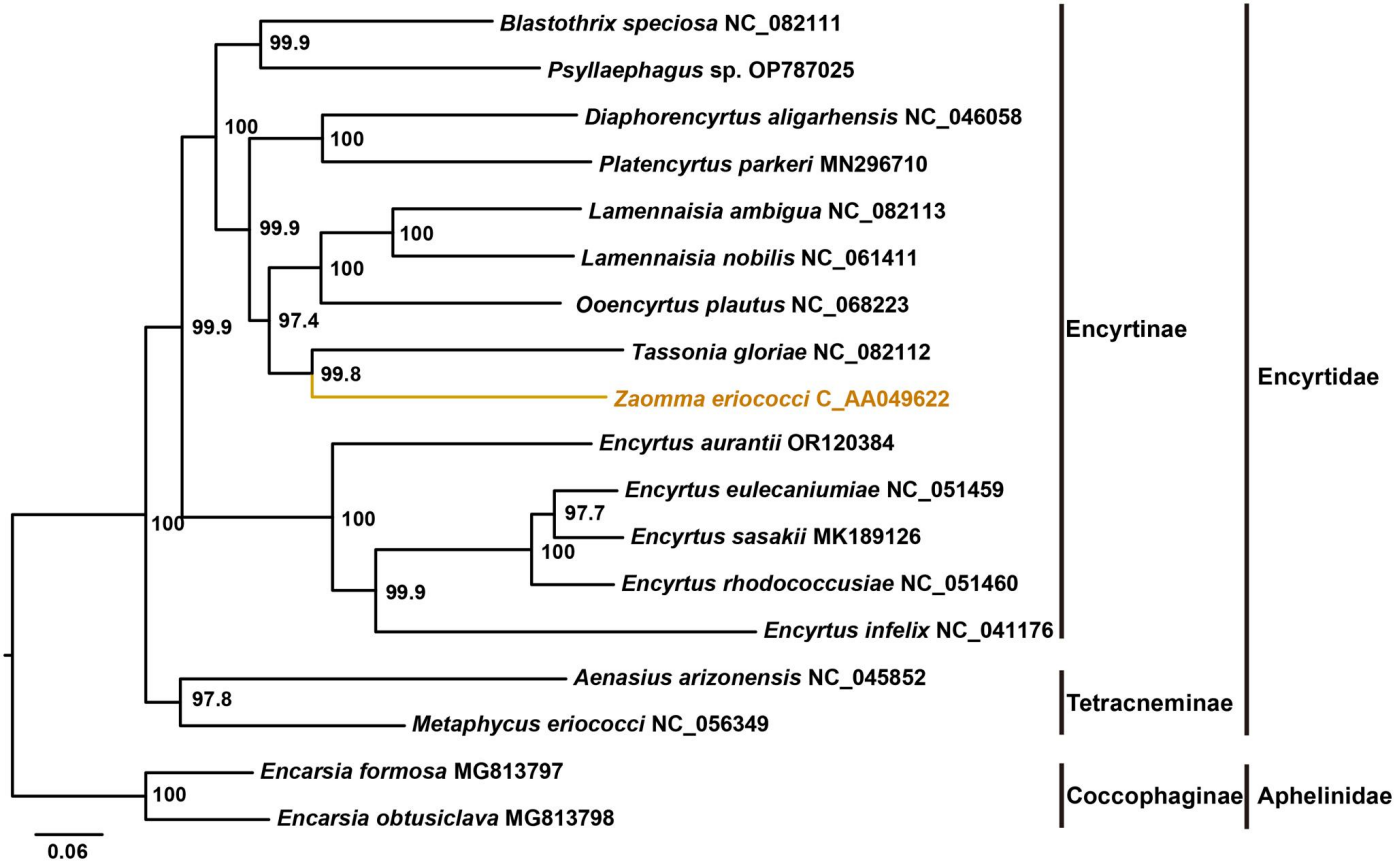
The maximum-likelihood (ML) phylogenetic tree of encyrtidae based on concatenated 13 mitochondrial protein coding genes. All species involved in the tree have scientific names with accession number on right side.

The ModelFinder analysis shown that the GTR + F + I + G4 model was the best-fit model. The maximum-likelihood phylogenetic tree based on the concatenation alignment of 13 PCGs, supported the sister relationship between Encyrtinae and Tetracneminae (Zhou et al. 2021; Xing et al. 2022) with high support values (>97%). The phylogeny also confirmed the placement of *Z. eriococci* within the subfamily Encyrtinae, indicating its close relationship to *Tassonia gloriae* (Figure 3).

## Discussion and conclusion

Approximately 5,000 species have been described in the family Encyrtidae (Noyes 2018). However, fewer than 50 complete mitochondrial genomes have been deposited in the GenBank database. In this study, we assembled and annotated the entire mitochondrial genome of *Z. eriococci* which it is the first for genus *Zaomma*. We described the structural features of the mitochondrial genome and its phylogenetic position within the Encyrtidae. The complete mitochondrial genome was 15,648 bp in length, with an AT content of 97.87%. The mitochondrial genome of *Z. eriococci* exhibits a novel gene rearrangement. Phylogenetic analysis supported the placement of *Z. eriococci* in the subfamily Encyrtinae, closely related to *T. gloriae*. These results will contribute to enhancing the mitochondrial genomic data for the family Encyrtidae.

## Supplementary Material

Supplemental Material

## Data Availability

The data supporting the findings of this study are openly available in the GenBase of CNCB at https://www.cncb.ac.cn/ under the accession number C_AA049622. The associated BioProject, BioSample and SRA numbers are PRJCA021546, SAMC3194389, and CRR958654, respectively.
