# Supplementary material for: The complete mitochondrial genome of Zaomma eriococci (hymenoptera: encyrtidae)
Source: Mitochondrial DNA B Resour. 2024 Jun 12;9(6):707–10. doi: 10.1080/23802359.2024.2351539 (PMC11172251; doi:10.1080/23802359.2024.2351539)
Supplement: Supplemental Material [file TMDN_A_2351539_SM6490.docx]

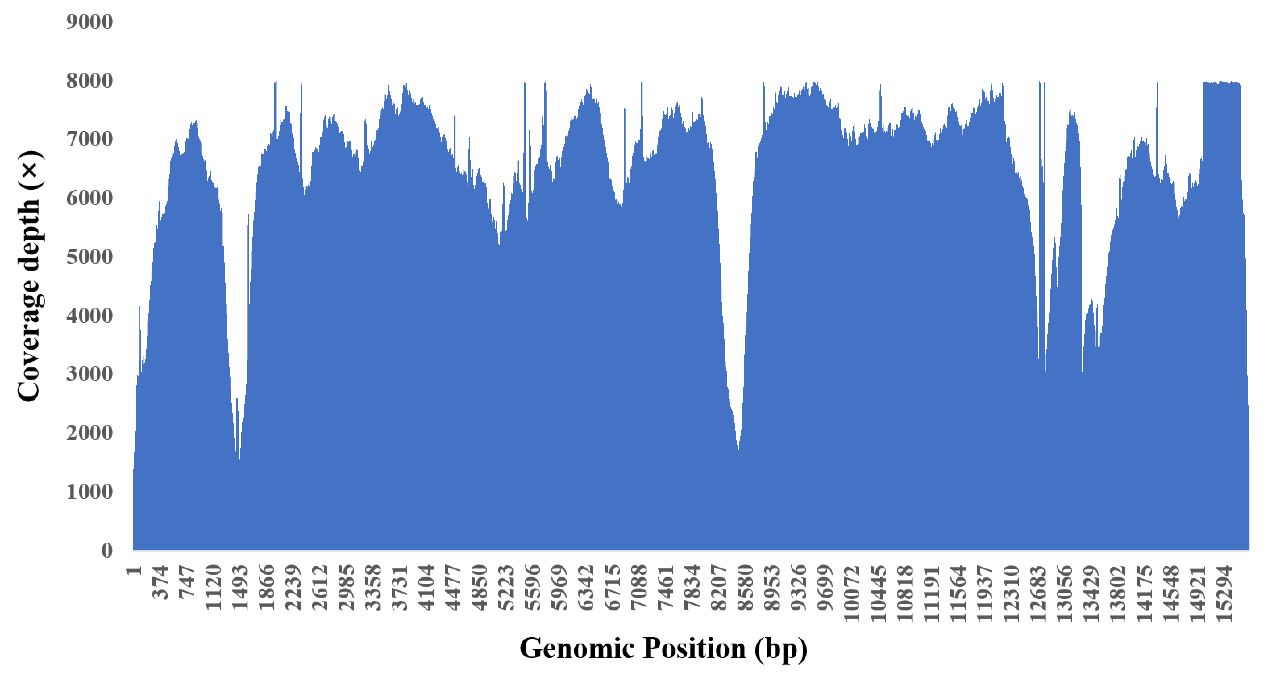


**Figure S1**: Coverage Depth of the *Zaomma eriococci* mitochondrial genome. The X and Y axes represent the nucleotide position of the *Z. eriococci* mitochondrial genome and the corresponding coverage depth, respectively. The minimum and maximum mapping depth are 870× and 7995×, respectively.

**Table S1**. List of all 18 species, GenBank or GenBase accession numbers and references for sequences used to construct phylogenetic trees (Figure 3).

| **Species** | **Family** | **Subfamily** | **Accession** | **Reference** |
| --- | --- | --- | --- | --- |
| *Blastothrix speciosa* | Encyrtidae | Encyrtinae | NC_082111 | Unpublished (submitted by Zhang,C.H. and Zu,G.H.) |
| *Psyllaephagus sp.* | Encyrtidae | Encyrtinae | OP787025 | Unpublished (submitted by Qin,Z. and Shi,W.) |
| *Diaphorencyrtus aligarhensis* | Encyrtidae | Encyrtinae | NC_046058 | Du et al. 2019 |
| *Platencyrtus parkeri* | Encyrtidae | Encyrtinae | MN296710 | Zhang et al. 2019 |
| *Lamennaisia ambigua* | Encyrtidae | Encyrtinae | NC_082113 | Unpublished (submitted by Wang,Y et al.) |
| *Lamennaisia nobilis* | Encyrtidae | Encyrtinae | NC_061411 | Unpublished (submitted by Wang,Y.) |
| *Ooencyrtus plautus* | Encyrtidae | Encyrtinae | NC_068223 | Xing et al. 2022 |
| *Tassonia gloriae* | Encyrtidae | Encyrtinae | NC_082112 | Unpublished (submitted by Zhang,C.H. and Zu,G.H.) |
| *Zaomma eriococci* | Encyrtidae | Encyrtinae | C_AA049622 | This study |
| *Encyrtus aurantii* | Encyrtidae | Encyrtinae | OR120384 | Unpublished (submitted by Zhang,C.H. and Zu,G.H.) |
| *Encyrtus eulecaniumiae* | Encyrtidae | Encyrtinae | NC_051459 | Unpublished (submitted by Xiong,M. and Zhang,Y.Z.) |
| *Encytus sasakii* | Encyrtidae | Encyrtinae | MK189126 | Unpublished (submitted by Xiong,M. and Zhang,Y.Z.) |
| *Encyrtus rhodococcusiae* | Encyrtidae | Encyrtinae | NC_051460 | Unpublished (submitted by Xiong,M. and Zhang,Y.Z.) |
| *Encyrtus infelix* | Encyrtidae | Encyrtinae | NC_041176 | Xiong et al. 2018 |
| *Aenasius arizonensis* | Encyrtidae | Tetracneminae | NC_045852 | Ma et al. 2019 |
| *Metaphycus eriococci* | Encyrtidae | Encyrtinae | NC_056349 | Zhou et al. 2021 |
| *Encarsia formosa* | Aphelinidae | Coccophaginae | MG813797 | Zhu et al. 2018 |
| *Encarsia obtusiclava* | Aphelinidae | Coccophaginae | MG813798 | Zhu et al. 2018 |

Du, Y., Song, X., Liu, X., and Zhong, B. 2019. Mitochondrial genome of *Diaphorencyrtus aligarhensis* (Hymenoptera: Chalcidoidea: Encyrtidae) and phylogenetic analysis. Mitochondrial DNA B Resour 4(2): 3190-3191. doi:10.1080/23802359.2019.1667913.

Ma, Y., Zheng, B.-y., Zhu, J.-c., Tang, P., and Chen, X.-x. 2019. The mitochondrial genome of *Aenasius arizonensis* (Hymenoptera: Encyrtidae) with novel gene order. Mitochondrial DNA B Resour 4(1): 2023-2024. doi:10.1080/23802359.2019.1617052.

Xing, Z.P., Liang, X., Wang, X., Hu, H.Y., and Huang, Y.X. 2022. ﻿Novel gene rearrangement pattern in mitochondrial genome of *Ooencyrtus plautus* Huang & Noyes, 1994: new gene order in Encyrtidae (Hymenoptera, Chalcidoidea). Zookeys 1124(1-21. doi:10.3897/zookeys.1124.83811.

Xiong, M., Zhou, Q.-S., and Zhang, Y.-Z. 2018. The complete mitochondrial genome of *Encyrtus* *infelix* (Hymenoptera: Encyrtidae). Mitochondrial DNA B Resour 4(1): 114-115. doi:10.1080/23802359.2018.1537727.

Zhang, Y.Z., Xiong, M., Zhou, Q.S., Jiang, G.C., and Zhu, C.D. 2019. The mitochondrial genome of *Platencyrtus parkeri* Feriere (Hymenoptera: Encyrtidae). Mitochondrial DNA B Resour 4(2): 3479-3481. doi:10.1080/23802359.2019.1674729.

Zhou, Q.-S., Xiong, M., Luo, A., Zhang, Y.-Z., and Zhu, C.-D. 2021. The complete mitochondrial genome of *Metaphycus eriococci* (Timberlake) (Hymenoptera: Encyrtidae). Mitochondrial DNA B Resour 6(2): 550-552. doi:10.1080/23802359.2021.1872450.

Zhu, J.C., Tang, P., Zheng, B.Y., Wu, Q., Wei, S.J., and Chen, X.X. 2018. The first two mitochondrial genomes of the family Aphelinidae with novel gene orders and phylogenetic implications. Int J Biol Macromol 118(Pt A): 386-396. doi:10.1016/j.ijbiomac.2018.06.087.
